# Quality assessment of outcome reporting, publication characteristics and overall methodological quality in trials on synthetic mesh procedures for the treatment of pelvic organ prolapse for development of core outcome sets

**DOI:** 10.1007/s00192-021-04749-3

**Published:** 2021-04-08

**Authors:** Thais Regina de Mattos Lourenço, Vasilis Pergialiotis, Constantin M. Durnea, Abdullatif Elfituri, Jorge Milhem Haddad, Cornelia Betschart, Gabriele Falconi, Christiana Campani Nygaard, Stergios K. Doumouchtsis, CHORUS: An International Collaboration for Harmonising Outcomes, Research and Standards in Urogynaecology and Women’s Health

**Affiliations:** 1 Department of Obstetrics and Gynaecology, Urogynaecology Division, Hospital das Clínicas da Faculdade de Medicina da Universidade de São Paulo, São Paulo, SP Brazil; 2 Laboratory of Experimental Surgery and Surgical Research N S Christeas, Athens University Medical School, Athens, Greece; 3 Northwick Park Hospital, London North West University Healthcare NHS Trust, London, UK; 4 Department of Obstetrics and Gynaecology, Epsom & St Helier University Hospitals NHS Trust, London, UK; 5 Department of Gynecology, University Hospital of Zurich, Zurich, Switzerland; 6 Department of Obstetrics and Gynaecology, San Bortolo Hospital, Vicenza, Italy; 7 Department of Obstetrics and Gynaecology, Hospital São Lucas, Pontifícia Universidade Católica do Rio Grande do Sul, Porto Alegre, Brazil; 8 St George’s University of London, London, UK; 9 American University of the Caribbean, School of Medicine, Pembroke Pines, Florida USA

**Keywords:** Pelvic organ prolapse, Mesh prolapse surgery, Core outcome sets, Synthetic mesh

## Abstract

**Introduction and hypothesis:**

Variations in outcome measures and reporting of outcomes in trials on surgery for pelvic organ prolapse (POP) using synthetic mesh have been evaluated and reported. However, the quality of outcome reporting, methodology of trials and their publication parameters are important considerations in the process of development of Core Outcome Sets. We aimed to evaluate these characteristics in randomized controlled trials on surgery for POP using mesh.

**Methods:**

Secondary analysis of randomized controlled trials on surgical treatments using synthetic mesh for POP previously included in a systematic review developing an inventory of reported outcomes and outcome measures. The methodological quality was investigated with the modified Jadad criteria. Outcome reporting quality was evaluated with the MOMENT criteria. Publication parameters included publishing journal, impact factor and year of publication.

**Results:**

Of the 71 previously reviewed studies published from 2000 to 2017, the mean JADAD score was 3.59 and the mean MOMENT score was 4.63. Quality of outcomes (MOMENT) was related to methodological quality (JADAD) (rho = 0.662; *p* = 0.000) and to year of publication (rho = 0.262; *p* = 0.028).

**Conclusions:**

Methodological quality and outcome reporting quality appear correlated. However, publication characteristics do not have strong associations with the methodological quality of the studies. Evaluation of the quality of outcomes, methodology and publication characteristics are all an indispensable part of a staged process for the development of Core Outcome and Outcome Measure Sets.

**Supplementary Information:**

The online version contains supplementary material available at 10.1007/s00192-021-04749-3.

## Introduction

The use of synthetic mesh for pelvic organ prolapse (POP) surgical treatment has recently decreased because of concerns around patient safety. These concerns have been the subject of extensive debates [1], and synthetic meshes for transvaginal POP repair were reclassified by the FDA as high-risk devices [1–4]. A high level of evidence on efficacy and safety through systematic reviews and meta-analyses is warranted to aid clinicians, policy makers and women in choices of treatment for pelvic organ prolapse. However, published data are frequently conflicting possibly on account of arbitrary outcome selection and reporting. Current research evidence is of variable quality and methodology and hence robust practice recommendations are lacking because of limitations in research evidence. Studies have reported on variable outcomes, and comparisons among several trials are not possible because of this heterogeneity [5].

The process of the development of a Core Outcome Set (COS) includes in-depth evaluation of the selection of outcomes reported in primary trials. Trials often use variable methods, and selection of outcomes varies because of authors’ expertise, research priorities and objectives, ethical issues and other factors. Frequently, trials on the same treatment intervention have been designed with different methods and study objectives and have been published in various journals (subspecialized, specialized, general journals) over the years. Our previous systematic review developed the inventory of reported outcomes and outcomes measures as a first step in the process of developing relevant COS based on established standards and following well-defined study protocols and high-quality methods [6]. Our systematic reviews on outcome reporting in trials evaluating surgical treatments in different prolapse procedures, incontinence and childbirth trauma [5, 7–12] included assessments of associations between outcome reporting quality, methodological quality and publication characteristics.

Evaluation of the quality outcome reporting and its associations with methodological quality might provide useful evidence in developing a core outcome set for this area of research and possibly provide valuable guidance and directions for future studies.

The aim of this study was to evaluate methodological quality and outcome quality in trials on surgical treatment of POP using synthetic mesh and assess the associations of methodological quality and outcome quality with publication characteristics of the trials including year of publication and journal impact factor.

## Materials and methods

This review is part of CHORUS (An International Collaboration for Harmonizing Outcomes, Research and Standards in Urogynecology and Women’s Health, i-chorus.org). This study was a secondary analysis of data that were part of a recently published systematic review [5]. Ethical approval was not required for this study, as this is a secondary analysis of data included in a previously published systematic review.

**Fig. 1:**
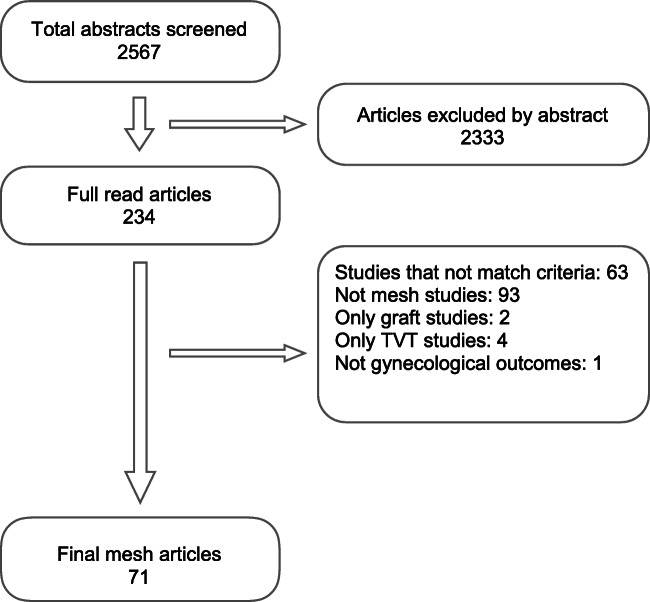
Randomized controlled trials (RCTs) using synthetic meshes for any type of pelvic prolapse were included

The search strategy was described in the original study in accordance with PRISMA guidelines. We used the following keywords and MeSH terms: management, repair, operation and pelvic organ prolapse in the Cochrane, EMBASE, Medline and Scopus databases from inception until September 2017. Randomized controlled trials (RCTs) using synthetic meshes for any type of pelvic prolapse were included (Fig. 1). We excluded retrospective studies, literature reviews, case reports and non-randomized studies. The studies selected are listed in Appendix 1.

The methodological quality of the included trials was evaluated using the modified Jadad score. This is a 5-point scale that evaluates randomization, adequate method for randomization; blinded trial described; adequate method for blinding and if the trial accounts for the patients selected [13]. The outcome quality was assessed using the MOMENT criteria (Management of otitis media with effusion in cleft palate score system), in a 6-point scale. Areas included in the scoring system are stating a primary outcome; explaining if the primary outcome is defined for reproducible measures; stating a secondary outcome; reporting if the secondary outcome is defined as for reproducible measures; explaining if the choice of outcome and if the methods used are designed to improve appropriately the quality of measures [14]. High-quality studies were considered those that reached score ≥ 4 based on these criteria. All assessments were undertaken independently by two assessors in line with our previous studies and similar research [7–12]. In cases of disagreement, a third assessor reviewed the scores and provided additional scores. The final scores were calculated and reviewed by the senior investigator.

Year and journal of publication and journal impact factor (IF) were retrieved and documented according to Thomson Reuters’ (NY, USA) citation reports for obstetrics and gynecology. Statistical analysis was undertaken using SPSS statistical software (IBM Corp., USA). Association among methodological quality, outcome quality, year of publication and journal’s impact factor was calculated by non-parametric correlation (Spearman’s correlation). Statistical significance was defined as *p* < 0.05.

## Results

We reviewed and assessed the 71 RCTs previously included in our systematic review.

**Fig. 2:**
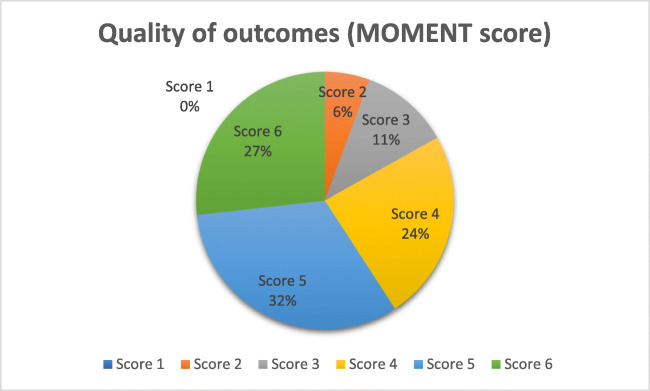
Distribution of scores on quality of outcome reporting (MOMENT scores)

**Fig. 3:**
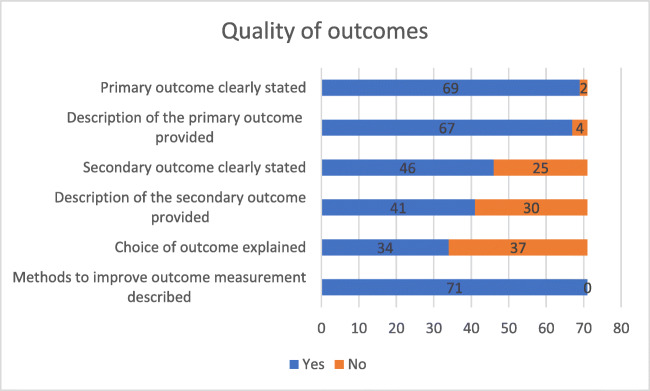
Number of studies satisfying the specific statements (yes or no) on quality of outcomes’ assessment

Methodological quality, outcome quality and publication parameters are presented in Table 1. Year of publication ranged from 2000 to 2017 and follow-up interval ranged from 1.5 to 74 months. The mean Jadad and MOMENT scores were 3.59 and 4.63. Figure 2 shows the quality of outcome (MOMENT) distribution for the total score, and Fig. 3 shows the number of studies scored according to each statement. Fifty-nine studies (83%) were classified as high-quality outcome reporting, presenting score 4, 5 or 6.

**Table 1:** Quality of studies

Author	Year	Journal	IF^a^	Jadad	MOMENT
Altman et al.	2011	New England Journal of Medicine	29.1	4	5
Anger et al.	2014	Obstetrics and Gynecology	4.76	2	5
Barber et al.	2009	Obstetrics and Gynecology	4.69	3	4
Bradley et al.	2007	American Journal of Obstetrics and Gynecology	4.45	3	4
Bradley et al.	2008	American Journal of Obstetrics and Gynecology	4.45	3	4
Carey et al.	2009	BJOG: An International Journal of Obstetrics and Gynecology	4.64	3	5
Chmielewski et al.	2011	American Journal of Obstetrics and Gynecology	5.34	4	4
Choe et al.	2000	Journal of Urology	2.64	2	3
Constantini et al.	2016	Journal of Urology	4.68	5	6
Constantini et al.	2007	European Urology	5.96	3	3
Coolen et al.	2017	International Urogynecology Journal	2.078	3	6
Culligan et al.	2005	Obstetrics and Gynecology	4	5	6
Culligan et al.	2013	Obstetrics and Gynecology	4.78	5	6
Cundiff et al.	2008	American Journal of Obstetrics and Gynecology	4.98	3	4
de Tayrac et al.	2013	International Urogynecology Journal	2.53	3	5
Delroy et al.	2013	International Urogynecology Journal	2.45	5	6
Dias et al.	2016	Neurourology and Urodynamics	2.48	5	6
Ek et al.	2013	International Urogynecology Journal	2.53	2	4
Ek et al.	2010	Neurourology and Urodynamics	3.01	5	4
El-Nazer et al.	2012	American Journal of Obstetrics and Gynecology	1.56	5	5
Farthmann et al.	2013	International Urogynecology Journal	2.45	3	3
Freeman et al.	2013	International Urogynecology Journal	2.53	5	6
Glazener et al.	2016	Trials	N/A	4	6
Glazener et al.	2017	Health Technology Assessment	N/A	4	6
Gupta et al.	2014	South African Journal of Obstetrics & Gynecology	0.23	3	4
Halaska et al.	2012	American Journal of Obstetrics and Gynecology	5.32	3	5
Heinonen et al.	2011	European Journal of Obstetrics & Gynecology and Reproductive Biology	2.58	3	5
Hiltunen et al.	2007	Obstetrics and Gynecology	4.45	3	4
Iglesia et al.	2010	Obstetrics and Gynecology	4.98	5	6
Lakeman et al.	2011	Journal of Sexual Medicine	3.67	3	4
Lamblin et al.	2014	International Urogynecology Journal	2.45	3	5
Lopes et al.	2010	International Urogynecology Journal	2.66	3	3
Madhuvrata et al.	2011	Journal of Obstetrics and Gynecology	0.75	5	5
Maher et al.	2003	American Journal of Obstetrics and Gynecology	3.59	3	5
Maher et al.	2012	American Journal of Obstetrics and Gynecology	5.32	5	6
Maher et al.	2011	American Journal of Obstetrics and Gynecology	5.34	5	6
Menefee et al.	2011	Obstetrics and Gynecology	5.34	5	6
Milani et al.	2011	Journal of Sexual Medicine	3.67	3	6
Natale et al.	2009	International Urogynecology Journal	2.84	3	5
Nieminen et al.	2010	American Journal of Obstetrics and Gynecology	4.98	3	4
Nieminen et al.	2008	International Urogynecology Journal	2.51	3	2
Noé et al.	2013	Archives of Gynecology and Obstetrics	1.63	3	2
Noé et al.	2015	Journal of Endourology	2.09	3	4
Nygaard et al.	2008	American Journal of Obstetrics and Gynecology	4.7	2	4
Nygaard et al.	2013	JAMA - Journal of the American Medical Association	13.59	3	4
Paraiso et al.	2011	Obstetrics and Gynecology	5.34	5	6
Park et al.	2013	International Urogynecology Journal	2.45	3	5
Qatawneh et al.	2013	Gynecological Surgery	0.46	3	5
Rahmanou et al.	2015	International Urogynecology Journal	1.83	3	5
Rane et al.	2004	Australian and New Zealand Journal of Obstetrics and Gynecology	0.87	5	5
Rondini et al.	2015	International Urogynecology Journal	2.17	3	4
Roovers et al.	2005	Neurourology and Urodynamics	3.23	3	5
Roovers et al.	2004	BJOG: An International Journal of Obstetrics and Gynecology	3.01	3	5
Rudnicki et al.	2015	BJOG: An International Journal of Obstetrics and Gynecology	2.9	3	3
Rudnicki et al.	2014	BJOG: An International Journal of Obstetrics and Gynecology	2.9	3	5
Sand et al.	2001	American Journal of Obstetrics and Gynecology	2.72	3	4
Silveira et al.	2014	International Urogynecology Journal	2.17	3	5
Shi et al.	2017	Medical Science Monitor	N/A	2	3
Sivaslioglu et al.	2008	International Urogynecology Journal	2.79	3	2
Svabik et al.	2014	Ultrasound in Obstetrics and Gynecology	4.5	4	5
Tamanini et al.	2015	Journal of Urology	4.68	4	5
Tamanini et al.	2013	International Braz J Urol: official journal of the Brazilian Society of Urology	1.24	4	5
Tamanini et al.	2013	International Braz J Urol: official journal of the Brazilian Society of Urology	1.24	4	5
Tan-Kim et al.	2014	International Urogynecology Journal	2.17	5	6
Turgal et al.	2013	European Journal of Obstetrics & Gynecology and Reproductive Biology	2.4	3	2
Visco et al.	2008	International Urogynecology Journal and Pelvic Floor Dysfunct.	2.51	5	4
Vollebregt et al.	2012	Journal of Sexual Medicine	3.67	5	6
Vollebregt et al.	2011	BJOG: An International Journal of Obstetrics and Gynecology	2.96	5	6
Weber et al.	2001	American Journal of Obstetrics and Gynecology	2.72	2	3
Withagen et al.	2011	Obstetrics and Gynecology	5.34	5	6
Yuk et al.	2012	Journal of Minimally Invasive Gynecology	2.1	3	3

^a^Journal’s impact factor

Primary and secondary outcomes were clearly stated in 69 (97%) and 46 (64%) of the studies. Only 34 studies (47%) provided a rationale for the choice of outcomes but all of them showed methods to improve the outcome measurement described.

A non-parametric correlation revealed that the outcome reporting quality was positively correlated to the methodological quality of the trial (rho = 0.662; *p* = 0.000) and to the year of publication (rho = 0.262; *p* = 0.028). Methodological quality does not appear to be influenced by year of publication (rho = 0.092; *p* = 0.444) or the journal’s impact factor (rho = 0.100; *p* = 0.417) (Table 2).

**Table 2:** – Univariate correlation on publication characteristics, JADAD score and MOMENT score

	MOMENT score			JADAD			IF^b^		
	Spearman’s Rho	*p*	*N*	Spearman’s Rho	*p*	*N*	Spearman’s Rho	*p*	*N*
Year^a^	0,262	0,028	71	0,920	0,444	71	–0,320	0,008	68
IF^b^	0,162	0,187	68	0,100	0,417	68	–	–	–
JADAD	0,662	0,000	71	–	–	–	–	–	–

^a^Year of publication

^b^Journal’s impact factor

*N *﻿number

## Discussion

In our study,outcome reporting quality ( MOMENT criteria) demonstrated a positive correlation to methodological quality (Jadad score) and to year of publication. The quality of outcome reporting has improved in more recently published trials. However, methodological quality did not have an association with year of publication or the journal’s impact factor.

Not surprisingly, methodological quality and outcome reporting quality appear correlated possibly because of the overall study design process and the overall quality of a research protocol. However, such “grading” of the overall quality of a study does not necessarily translate into a publication in a journal with a higher impact factor. This observation highlights the need for harmonization of the quality of methodology and of the reported outcomes and possibly the development of set criteria in research protocols that may assist journal editors in the peer review publication process.

The methodological quality and quality of reported outcomes as well as publication characteristics should be taken into consideration during the process of development of core outcome sets.

One of the strengths of our systematic review is to be the first one to our knowledge evaluating the methodological quality as well as outcome reporting quality in trials using mesh for the treatment of pelvic organ prolapse. We followed a well-established methodology in a standardized manner in order to provide unbiased and objective evaluation of the above-mentioned parameters of the published trials. Another strength of our study was the independent assessments that were undertaken and the process of review and consensus around the final scores of the different domains and items.

However, a number of limitations warrant caution in the interpretation of our results.

We evaluated a highly selective cohort of studies leaving out studies that were non-randomized and with different methodologies. Nevertheless, inclusion of a wider variety of studies would be likely to demonstrate wider variations and accentuate our findings. Furthermore, our findings are based on the instruments used and their inherent limitations. Some studies with higher quality protocols but with suboptimal descriptions of these protocol in the published text may have received lower scores than deserved.

Finally, on reviewing the correlation of the quality of the trial and the journal impact factor, we should take into consideration that the choice of the journal a study was published in can be influenced by many factors not necessarily associated directly with the quality of the study. For example, a presentation of a study at a conference may be rewarded by an offer of a fast track review of the paper in the journal associated to the society organizing the conference. Some authors may also select a journal to submit based on personal preference, influenced by factors such as journal loyalty of the author, previous or current association with the journal’s editorial board or other factors. Hence, this correlation between quality of a trial and journal of publication should always be interpreted with caution for such biases that cannot be weighed.

In the studies included in this evaluation, methodological quality of trials was positively associated with outcome quality but was not strongly associated to year of publication and the journal’s impact factor. The instruments and methodology described, already widely used in many fields of medical research, in gynecology, obstetrics and urogynecology, were applied to analyze in a standardized way important parameters of published research [5]. This assessment is in our opinion a fundamental prerequisite in the process of developing a high quality COS in line with the standards established by the COMET, COSMIN and CROWN initiatives.

Nevertheless, we believe that this study may also provide invaluable guidance for improving better selection of outcomes and measurement tools, outcome reporting, research methods and publication strategies for future research in this area. Harmonized methodology and outcome selection and reporting may improve the comparability of primary research, which in turn may inform robust meta-analyses and eventually improve clinical practice.


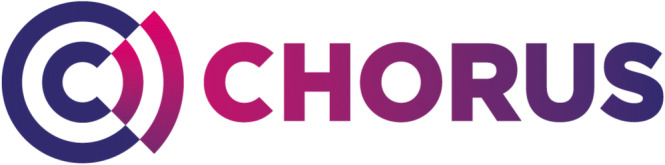


## Supplementary Information

ESM 1 (DOC 52 kb)
