# Supplementary material for: Quality assessment of outcome reporting, publication characteristics and overall methodological quality in trials on synthetic mesh procedures for the treatment of pelvic organ prolapse for development of core outcome sets
Source: Int Urogynecol J. 2021 Apr 8;32(11):2913–9. doi: 10.1007/s00192-021-04749-3 (PMC8536576; doi:10.1007/s00192-021-04749-3)
Supplement: Supplementary file 1 — (DOC 52 kb) [file 192_2021_4749_MOESM1_ESM.doc]

**References for selected studies with synthetic mesh**

**Vaginal mesh**

Altman D, Vayrynen T, Engh ME, Axelsen S, Falconer C. Anterior colporrhaphy versus transvaginal mesh for pelvic-organ prolapse. N Engl J Med. 2011 May 12;364(19):1826-36 PMID [21561348] DOI: 10.1056/NEJMoa1009521

Ek M, Altman D, Gunnarsson J, Falconer C, Tegerstedt G. Clinical efficacy of a trocar-guided mesh kit repairing lateral defects. Int Urogynecol J. 2013 Feb;24(2):249-54 PMID [22707003] DOI: 10.1007/s00192-012-1833-9

Carey M, Higgs P, Goh J, Leong A, Krause H, Cornish A. Vaginal repair with mesh versus colporrhaphy for prolapse: a randomised controlled trial. BJOG. 2009 Sep;116(10):1380-6 PMID [19583714] DOI: 10.1111/j.1471-0528.2009.02254.x

Choe JM, Ogan K, Battino BS. Antimicrobial mesh versus vaginal wall sling: a comparative outcomes analysis. J Urol. 2000 Jun;163(6):1829-34 PMID [ 10799192]

Delroy CA, Castro RA, Dias MM, Feldner Jr PC, Bortolini MAT, Girão MJBC et al. The use of transvaginal synthetic mesh for anterior vaginal wall prolapse repair: a randomized controlled trial. Int Urogynecol J. 2013 Nov;24(11):1899-907 PMID [23632800] DOI: 10.1007/s00192-013-2092-0

Dias MM, Castro RA, Bortolini MAT, Delroy CA, Martins PCF, Girão MJBC et al. Two-years results of native tissue versus vaginal mesh repair in the treatment of anterior prolapse according to different success criteria: a randomized controlled trial. Neurourol Urodyn. 2016 Apr;35(4):509-14 PMID [25810682] DOI: 10.1002/nau.22740

Ek M, Tegerstedt G, Falconer C, Kjaeldgaard A, Rezapour M, Rudnicki M et al. Urodynamic assessment of anterior vaginal wall surgery: a randomized comparison between colporraphy and transvaginal mesh. Neurourol Urodyn. 2010 Apr;29(4):527-31 PMID [19731311] DOI: 10.1002/nau.20811

El-Nazer MA, Gomaa IA, Madkour WAI, Swidan KH, El-Etriby MA. Anterior colporrhaphy versus repair with mesh for anterior vaginal wall prolapse: a comparative clinica study. Arch Gynecol Obstet. 2012 Oct;286(4):965-72 PMID [22648445] DOI: 10.1007/s00404-012-2383-6

Farthmann J, Watermann D, Niesel A, Funfgeld C, Kraus A et al. Lower exposure rates of partially absorbable mesh compared to nonabsorbable mesh for cystocele treatment: a 3-year follow-up of a prospective randomized trial. Int Urogynecol J. 2013 May;24(5):749-58 PMID [22930216] DOI: 10.1007/s00192-012-1929-2

Glazener C, Breeman S, Elders A, Hemming C, Cooper K, Freeman R et al. Clinical effectiveness and cost-effectiveness of surgical options for the management of anterior and/or posterior vaginal wall prolapse: two randomised controlled trials within a comprehensive cohort study - results from the PROSPECT Study. Health Technol Assess. 2016 Dec;20(95):1-452 PMID [28052810] DOI: 10.3310/hta20950

Glazener CMA, Breeman S, Elders A, Hemming C, Cooper KG, Freeman RM et al. Mesh, graft or standard repair for women having primary transvaginal anterior or posterior compartment prolapse surgery: two parallel-group, multicentre, randomised, controlled trials (PROSPECT). Lancet. 2017 Jan 28;389(10067):381-392. PMID [28010989] DOI: 10.1016/S0140-6736(16)31596-3

Gupta B, Vaid NB, Suneja A, Guleria K, Jain S. Anterior vaginal prolapse repair: a randomised trial of traditional anterior colporrhaphy and self-tailored mesh repair. SAJOG 2014 Aug;20(2):47-50

Halaska M, Maxova K, Sottner O, Svabik K, Mlcoch M, Kolarik D et al. A multicenter, randomized, prospective, controlled study comparing sacrospinous fixation and transvaginal mesh in the treatment os posthysterectomy vaginal vault prolapse. Am J Obstet Gynecol. 2012 Oct;207(1):301.e1-7 PMID [23021692] DOI: 10.1016/j.ajog.2012.08.016

Heinonen PK, Nieminen K. Combined anterior vaginal wall mesh with sacrospinous ligament fixation or with posterior intravaginal slingplasty for uterovaginal or vaginal vault prolapse. Eur J Obstet Gynecol Reprod Biol. 2011 Aug;157(2):230-3 PMID [21561703] DOI: 10.1016/j.ejogrb.2011.03.031

Hiltunen R, Nieminen K, Takala T, Heiskanen E, Merikari M, Niemi K et al. Low-weight polypropylene mesh for anterior vaginal wall prolapse: a randomized controlled trial. Obstet Gynecol. 2007 Aug;110(2 Pt 2):455-62 PMID [17666627] DOI: 10.1097/01.AOG.0000261899.87638.0a

Nieminen K, Hiltunen R, Heiskanen E, Takala T, Niemi K, Merikari M et al. Symptom resolution and sexual function after anterior vaginal wall repair with or without polypropylene mesh. Int Urogynecol J Pelvic Floor Dysfunct. 2008 Dec;19(12):1611-6 PMID [18716704] DOI: 10.1007/s00192-008-0707-7

Nieminen K, Hiltunen R, Takala T, Heiskanen E, Merikari M, Niemi K et al. Outcomes after anterior vaginal wall repair with mesh: a randomized, controlled trial with a 3 years follow-up. Am J Obstet Gynecol. 2010 Sep;203(3):235.e1-8 PMID [20494332] DOI: 10.1016/j.ajog.2010.03.030

Iglesia CB, Sokol AI, Sokol ER, Kudish BI, Gutman RE, Peterson JL et al. Vaginal mesh for prolapse: a randomized controlled trial. Obstet Gynecol. 2010 Aug,116(1 Pt 1):293-303 PMID [20664388] DOI: 10.1097/AOG.0b013e3181e7d7f8

Lamblin G, Van-Nieuwenhuyse A, Chabert P, Lebail-Carval K, Moret S, Mellier G. A randomized controlled trial comparing anatomical and functional outcome between vaginal colposuspension and trnasvaginal mesh. Int Urogynecol J. 2014 Jul;25(7):961-70 PMID [24573358] DOI: 10.1007/s00192-014-2344-7

Lopes ED, Lemos NLBM, Carramão SS, Lunardelli JL, Ruano JMC, Aoki T et al. Transvaginal poypropylene mesh versus sacrospinous ligament fixation for the treatment of uterine prolapse: 1-year follow-up of a randoized controlled trial. Int Urogynecol J. 2010 Apr;21(4):389-94 PMID [19936588] DOI: 10.1007/s00192-009-1052-1

Madhuvrata P, Glazener C, Boachie C, Allahdin S, Bain C. A randomised controlled trial evaluating the use of polyglactin (Vicryl) mesh, polydioxanone (PDS) or polyglactin (Vicryl) sutures for pelvic organ prolapse surgery: outcomes at 2 years. J Obstet Gynaecol. 2011 Jul;31(5):429-35 PMID [21627429] DOI: 10.3109/01443615.2011.576282

Maher CF, Feiner B, DeCuyper EM, Nichols CJ, Hickey KV, O’Rourke P. Laparoscopic sacral colpopexy versus total vaginal mesh for vaginal vault prolapse: a randomized trial. Am J Obstet Gynecol. 2011 Apr;204(4):360.e1-7. PMID [21306698] DOI: 10.1016/j.ajog.2010.11.016

Maher CF, Connelly LB. Cost minimization analysis of laparoscopic sacral colpopexy and total vaginal mesh. Am J Obstet Gynecol. 2012 May;206(5):433.e1-7 PMID [22285170] DOI: 10.1017/j.ajog.2011.12.012

Menefee SA, Dyer KY, Lukacz EM, Simsiman AM, Luber KM, Nguyen JN. Colporrhaphy compared with mesh or graft-reinforced vaginal paravaginal repair for anterior vaginal wall prolapse: a rnadomized controlled trial. Obstet Gynecol. 2011 Dec;118(6):1337-44 PMID [22067717] DOI: 10.1097/AOG.ob013e318237edc4

Natale F, La Penna C, Padoa A, Agostini M, De Simone E, Cervigni M. A Prospective, randomized, controlled study comparing Gynemesh, a systetic mesh, and Pelvicol, a biologic graft, in the surgical treatment of recurrent cystocele. Int Urogynecol J. 2009 20:75-81 DOI: 10.1007/s00192-008-0732-6

Park HK, Paick SH, Lho YS, Choo GY, Kim HG, Choi J. Lack of effect of concomitant stage II cystocele repair on lower urinary tract symptoms and surgical outcome after tension-free vaginal tape procedure: randomized controlled trial. Int Urogynecol J. 2013 Jul;24(7):1123-6 PMID [23124692] DOI: 10.1007/s00192-012-1961-2

Qatawneh A, Al-Kazaleh F, Saleh S, Thekrallah F, Bata M, Sumreen I et al. Transvaginal cystocele repair using tension-free polypropylene mesh at the time of sacrospinous colpopexy for advanced uterovaginal prolapse: a prospective randomised study. Gynecol Surg. 2013;10:79-85 DOI: 10.1007/s10397-012-0759-0

Rudnicki M, Laurikainen E, Pogosean R, Kinne I, Jakobsson U, Teleman P. Anterior colporrhaphy compared with collagen-coated transvaginal mesh for anterior vaginal wall prolapse: a randomised controlled trial. BJOG 2014 Jan;121(1):102-10 PMID [24118844] DOI: 10.1111/1471-052812454

Rudnicki M, Laurikainen E, Pogosean R, Kinne I, Jakobsson U, Teleman P. A 3-year follow up after anterior colporraphy compared with collagen-coated transvaginal mesh for anterior vaginal wall prolapse: a randomised controlled trial. BJOG. 2016 Jan;123(1):136-42 PMID [26420345] DOI: 10.1111/1471-0528.13628

Sand PK, Koduri S, Lobel RW, Winkler HA, Tomezsko J, Culligan PJ et al. Prospective randomized trial of polygalactin 910 mesh to prevent recurrence of cystoceles and rectoceles. Am J Obstet Gynecol. 2001 Jun;184(7):1357-62 PMID [11408853] DOI: 10.1067/mob.2001.115118

Shi Y, Yu Y, Zhang X, Li Y. Trnasvaginak mesh and transanal resection to treat outlet obstruction constipation caused by rectocele. Med Sci Monit. 2017 Feb 1;23:598-605 PMID [28146137]

Silveira SRB, Haddad JM, Bella ZIKJ, Nastri F, Kawabata MGM, Carramão SS et al. Multicenter, randomized trial comapring native tissue repair and synthetic mesh repair for genitalprolapse surgical treatment. Int Urogynecol J. 2015 Mar;26(3):335-42 PMID [25199496] DOI: 10.1007/s00192-014-2501-z

Sivaslioglu AA, Unlubilgin E, Dolen I. A randomizeed comparison of polypropilene mesh surgery with site-specific surgery in the treatment of cystocele. Int Urogynecol J Pelvic Floor Dysfunct. 2008 Apr;19(4):467-71 PMID [17901910] DOI: 10.1001/s00192-007-0465-y

Svabik K, Martan A, Masata J, El-Haddad R, Hubka P. Comparison of vaginal mesh repair with sacrospinous vaginal colpopexy in the management of vaginal vault prolapse after hysterectomy in patients with levator ai avulsion: a randomized controlled trial. Ultrasound Obstet Gynecol. 2014 Apr;43(4):365-71 PMID [24615948] DOI: 10.1002/uog.13305

Tamanini JTN, Tamanini MMM, Castro RCOS, Feldner Jr PC, Castro RA, Sartori MGF et al. Treatment of anterior vaginal wall prolapse with and without polypropylene mesh: a prospective, randomized and controlled trial - Part I. Int Braz J Urol. 2013 Jul-Aug;39(4):519-30 PMID [24054380] DOI: 10.1590/S1677-5538.IBJU.2013.04.10

Tamanini JTN, Castro RCOS, Tamanini JM, Feldner Jr PC, Castro RA, Sartori MGF et al. Treatment of anterior vaginal wall prolapse with and without polypropylene mesh: a prospective, randomized and controlled trial - Part II. Int Braz J Urol. 2013 Jul-Aug;39(4):531-41 PMID [24054381] DOI: 10.1590/S1677-5538.IBJU.2013.04.11

Tamanini JTN, Castro RCOS, Tamanini JM, Castro RA, Sartori MGF, Girão MJBC. A prospective, randomized, controlled trial of the treatment of anterior vaginal wall prolapse: medium term followup. J Urol. 2015 Apr;193(4):1298-304 PMID [25305357] DOI: 10.1016/j.juro.2014.10.003

de Tayrac R, Cornille A, Eglin G, Guilbaud O, Mansoor A, Alonso S et al. Comparison between trans-obturator trans-vaginal mesh and traditional anterior colporrhaaphy in the treatment of anterior vaginal wall prolapse: results of a French RCT. Int Urogynecol J. 2013 Oct;24(10):1651-61 PMID [23512113] DOI: 10.1007/s00192-013-2075-1

Turgal M, Sivaslioglu A, Yildiz A, Dolen I. Anatomical and functional assessment of anterior colporrhaphy versus polypropylene mesh surgery in cistocele treatment. Eur J Obstet Gynecol Reprod Biol. 2013 Oct;170(2):555-8 PMID [23916584] DOI: 10.1016/j.ejogrb.2013.07.014

Vollebregt A, Fischer K, Gietelink D, van der Vaart CH. Primary surgical repair of anterior vaginal prolapse: a randomised trial comparing anatomical and functional outcome between anterior colporrhaphy and trocar-guided transobturator anterior mesh. BJOG. 2011 Nov;118(12):1518-27 PMID [21864325] DOI: 10.1111/j.1471-0528.2011.03082.x

Vollebregt A, Fischer K, Gietelink D, van der Vaart CH. Effects of vaginal prolapse surgery on sexuality in women and men; results from a RCT on repair with and without mesh. J Sex Med. 2012 Apr;9(4):1200-11 PMID [22321388] DOI: 10.1111/j.1743-6109.2011.02647.x

Weber AM, Walters MD, Piedmonte MR, Ballard LA. Anterior colporrhaphy: a randomized trial of three surgical techniques. Am J Obstet Gynecol. 2001 dec;185(6):1299-304 PMID [11744900] DOI: 10.1067/mob.2001.119081

Chmielewski L, Walters MD, Weber AM, Barber MD. Reanalysis of a randomized trial of 3 techniques of anterior colporrhaphy using clinically relevant definitions of sucess. Am J Obstet Gynecol. 2011 Jul;205(1):69.e1-8 PMID [21545996] DOI: 10.1016/j.ajog.2011.03.027

Withagen MI, Milani AL, den Boon J, Vervest HA, Vierhout ME. Trocar-guided mesh compared with conventional vaginal repair in recurrent prolapse: a randomized controlled trial. Obstet Gynecol. 2011 Feb;117(2 Pt 1):242-50 PMID [21252735] DOI: 10.1097/AOG/0b013e318203e6a5

Milani AL, Withagen MIJ, The HS, Nedelcu-van der Wijk I, Vierhout ME. Sexual function following trocar-guided mesh or vaginal native tissue repair in recurrent prolapse: a randomized controlled trial. J Sex Med. 2011 Oct;8(10):2944-53 PMID [21797984] DOI: 10.1111/j.1743-6109.2011.02392.x

Yuk JS, Jin CH, Yi KW, Kim T, Hur JY, Shin JH. Anterior transobturator polypropylene mesh in correction of cystocele: 2-point method vs 4-point method. J Minim Invasive Gynecol. 2012 Nov-De ;19(6):737-41 PMID [23084678] DOI: 10.1016/j.jmig.2012.08.769

**Abdominal mesh**

Anger JT, Mueller ER, Tarnay C, Smith B, Stroupe K, Rosenman A et al. Robotic compared with laparoscopic sacrocolpopexy: a randomized controlled trial. Obstet Gynecol. 2014 Jan;123(1):5-12 PMID [24463657] DOI: 10.1097/AOG.0000000000000006

Bradley CS, Nygaard IE, Brown MB, Gutman RE, Kenton KS, Whitehead WE et al. Bowel symptoms in women 1 year after sacrocolpopexy. AM J Obstet Gynecol. 2007 Dec;197(6):642.e1-8. PMID [18060963] DOI: 10.1016/j.ajog.2007.08.023

Barber MD, Brubaker L, Nygaard I, Wheeler II TL Schaffer J, Chen Z et al. Defining success after surgery for pelvic organ prolapse. Obstet Gynecol. 2009 Sep;114(3):600-9 PMID [19701041] DOI: 10.1097/AOG.0b013e3181b2b1ae

Bradley CS, Kenton KS, Richter HE, Gao V, Zyczynski HM, Weber AN et al. Obesity and outcomes after sacrocolpopexy. Am J Obstet Gynecol. 2008 Dec;199(6):690.e1-8 PMID [18845288] DOI: 10.1016/j.ajog.2008.07.030

Cundiff GW, Varner E, Visco AG, Zyczynski HM, Nager CW, Norton PA et al. Risk factors for mesh/suture erosion following sacral colpopexy. Am J Obstet Gynecol. 2008 Dec;199(6):688.e1-5 PMID [18976976] DOI: 10.1016/j.ajog.2008.07.029

Nygaard I, Handa VL, Brubaker L, Borello-France D, Wei J, Wells E et al. Changes in physical activity after abdominal sacrocolpopexy for advanced pelvic organ prolapse. Am J Obstet Gynecol. 2008 May;198(5):570.e1-5 PMID [18455536] DOI: 10.1016/j.ajog.2008.01.044

Nygaard I, Brubaker L, Zyczynski HM, Cundiff G, Richter H, Gantz M et al. Long-term outcomes following abdominal sacrocolpopexy for pelvic organ prolapse. JAMA. 2013 May 15;309(19):2016-24 PMID [23677313] DOI: 10.1001/jama.2013.4919

Visco AG, Brubaker L, Nygaard I, Richter HE, Cundiff G, Fine P et al. The role of preoperative urodynamic testing in stress-continent women undergoing sacrocolpopexy: the Colpopexy and Urinary Reduction Efforts (CARE) randomized surgical trial. Int Urogynecol J Pelvic Floor Dysfunct. 2008 May;19(5):607-14 PMID [18185903] DOI: 10.1007/s00192-007-0498-2

Coolen AWM, van Oudheusden AMJ, Mol BWJ, van Eijndhoven HWF, Roovers JWR, Bongers MY. Laparoscopic sacrocolpopexy compared with open abdominal sacrocolpopexy for vault prolapse repair: a randomized controlled trial. Int Urogynecol J. 2017 Oct;28(10):1469-1479 PMID [28417153] DOI: 10.1007/s00192-017-3296-5

Costantini E, Zucchi A, Giannantoni A, Mearini L, Bini V, Porena M. Must colposuspension be associated with sacropexy to prevent postoperative urinary incontinence? Eur Urol. 2007 Mar;51(3):788-94 PMID [17011699] DOI:10.1016/j.eururo.2006.08.034

Costantini E, Mearini L, Lazzeri M, Bini V, Nunzi E, di Biase M et al. Laparoscopic versus abdominal sacrocolpopexy: a randomized, controlled trial. J Urol. 2016 Jul;196(1):159-65 PMID [26780167] DOI: 10.1016/j.juro.2015.12.089

Culligan PJ, Blackwell L, Goldsmith LJ, Graham CA, Rogers A, Heit MH. A randomized controlled trial comparing fascia lata and synthetic mesh for sacral colpopexy. Obstet Gynecol. 2005 Jul;106(1):29-37 PMID [15994614] DOI: 10.1097/01.AOG.0000165824.62167.c1

Culligan PJ, Salamon C, Priestley JL, Shariati A. Porcine dermis compared with polypropylene mesh for laparoscopic sacrocolpopexy: a randomized controlled trial. Obstet Gynecol. 2013 Jan;121(1):143-51 PMID [23262939] DOI: 10.1097/AOG.0b013e31827558dc

Freeman RM, Pantazis K, Thomson A, Frappell J, Bombieri L, Moran P et al. A randomised controlled trial of abdominal versus laparoscopic sacrocolpopexy for the treatment of post-hysterectomy vaginal vault prolapse: LAS study. Int Urogynecol J. 2013 Mar;24(3):377-84 PMID [22864764] DOI: 10.1007/s00192-012-1885-x

Maher CF, Qatawneh AM, Dwyer PL, Carey MP, Cornish A, Schluter PJ. Abdominal sacral colpopexy or vaginal sacrospinous colpopexy for vaginal vault prolapse: a prospective randomized study. Am J Obstet Gynecol. 2004 Jan;190(1):20-6 PMID [14749629] DOI: 10.1016/j.ajog.2003.08.031

Maher CF, Feiner B, DeCuyper EM, Nichols CJ, Hickey KV, O’Rourke P. Laparoscopic sacral colpopexy versus total vaginal mesh for vaginal vault prolapse: a randomized trial. Am J Obstet Gynecol. 2011 Apr;204(4):360.e1-7. PMID [21306698] DOI: 10.1016/j.ajog.2010.11.016

Maher CF, Connelly LB. Cost minimization analysis of laparoscopic sacral colpopexy and total vaginal mesh. Am J Obstet Gynecol. 2012 May;206(5):433.e1-7 PMID [22285170] DOI: 10.1017/j.ajog.2011.12.012

Noé KG, Spuntrup C, Anapolski M. Laparoscopic pectopexy: a randomised comparative clinical trial of standard laparoscopic sacral colpo-cervicopexy to the new laparoscopic pectopexy. Short-term postoperative results. Arch Gynecol Obstet. 2013 Feb;287(2):275-80 PMID [22945837] DOI: 10.1007/s00404-012-2536-7

Noé KG, Schiermeier S, Alkatout I, Anapolski M. Laparoscopic pectopexy: a prospective, randomized, comparative clinical trial of standard laparoscopic sacral colpocervicopexy with the new laparoscopic pectopexy-postoperative results and intermediate-term follow-up in a pilot study. J Endourol. 2015 Feb;29(2):210-5 PMID [25350228] DOI: 10.1089/end.2014.0413

Paraiso MFR, Jelovsek JE, Frick A, Chen CCG, Barber MD. Laparoscopic compared with robotic sacrocolpopexy for vaginal prolapse: a randomized controlled trial. Obstet Gynecol. 2011 Nov;118(5):1005-13 PMID [21979458] DOI: 10.1097/AOG.0b013e318231537c

Rahmanou P, Price N, Jackson SR. Laparoscopic hysteropexy versus vaginal hysterectomy for the treatment of uterovaginal prolapse: a prospective randomized pilot study. Int Urogynecol J. 2015 Nov;26(11):1687-94 PMID[26142347] DOI: 10.1007/s00192-015-2761-2

Rane A, Lim YN, Withey G, Muller R. Magnetic resonance imaging findings following three different vaginal vault prolapse repair procedures: a randomised study. Aust N Z Obstet Gynaecol. 2004 Apr;44(2):135-9 PMID [15089837] DOI: 10.1111/j.1479-828X.2004.00186.x

Rondini C, Braun H, Alvarez J, Urzúa MJ, Villegas R, Wenzel C et al. High uterosacral vault suspension vc Sacrocolpopexy for treating apical defects: a randomized controlled trial with twelve months follow-up. Int Urogynecol J. 2015 Aug;26(8):1131-8 PMID [25910610] DOI: 10.1007/s00192-015-2666-0

Roovers JPWR, van der Vaart CH, van der Bom JG, Schagen van Leeuwen JH, Scholten PC, Heintz APM. A randomised controlled trial comparing abdominal and vaginal prolapse surgery: effects on urogenital function. BJOG. 2004 Jan;111(1):50-6. PMID [14687052]

Roovers JPWR, van der Bom JG, van der Vaart CH, Schagen van Leeuwen JH, Scholten PC et al. A randomized comparison of post-operative pain, quality of life, an physical performance during the first six weeks after abdominal or vaginal surgical correction of descensus uteri. Neurourol Urodyn. 2005;24(4):334-40 PMID [14924355] DOI: 10.1002/nau.20104

Lakerman MME, van der Vaart CH, Laan E, Roovers JPWR. The effect of prolapse surgery on vaginal sensibility. J Sex Med. 2011 Apr;8(4):1239-45 PMID [21235724] DOI: 10.1111/j.1743-6109.2010.02175.x

Tan-Kim J, Nager CW, Grimes CL, Luber KM, Lukacz ES, Brown HW et al. A randomized trial of vaginal mesh attachment techniques for minimally invasive sacrocolpopexy. Int Urogynecol J. 2015 May;26(5):649-56. PMID [25421934] DOI: 10.1007/s00192-014-2566-8
